# Mind wandering, poor sleep, and negative affect: a threefold vicious cycle?

**DOI:** 10.3389/fnhum.2024.1441565

**Published:** 2024-09-05

**Authors:** Juergen Fell

**Affiliations:** Department of Epileptology, University Hospital Bonn, Bonn, Germany

**Keywords:** mind wandering, affect, mood, emotion, sleep, depression, rumination, anxiety disorders

## Abstract

Mind wandering (MW) is intricately linked to sleep and affect, bearing clinical relevance for various psychiatric conditions, notably attention deficit/hyperactivity disorder, depression, and anxiety disorders. Most reviews concur that the relationship between disturbed sleep and negative affect is bidirectional. The directional relationships between MW propensity and disturbed sleep, as well as MW propensity and negative affect, are less clear. Therefore, this brief review aims to examine the limited studies that have directly explored temporally sequential relationships. These studies provide clear evidence for an impact of affect on MW and of MW on sleep, along with less unequivocal evidence for an influence of MW on affect and sleep on MW. Collectively, these individual reinforcement loops may constitute a threefold vicious cycle, which may contribute to the development and perpetuation of psychiatric disorders. Available data convincingly suggest an impact cycle in the direction “MW propensity → disturbed sleep → negative affect → MW propensity,” while evidence for the inverse impact cycle is less pronounced.

## 1 Introduction

The phenomenon of mind wandering (MW) holds significant implications for various psychiatric conditions, notably attention deficit/hyperactivity disorder (ADHD) [for an overview, see, e.g., Bozhilova et al. (2018)], depression [for an overview, see, e.g., Chaieb et al. (2022)], and anxiety disorders [for an overview, see, e.g., Fell et al. (2023)]. Existing research points to an increased amount of overall MW in individuals with ADHD and anxiety disorders, and waves of excessive MW are symptomatic of these conditions (Bozhilova et al., 2018; Fell et al., 2023). While the relationship between overall MW and depression is less conclusive (Chaieb et al., 2022), it has been observed that MW shifts towards more negatively valenced, self-and past-related contents in depression (Hoffmann et al., 2016).

Various definitions of the term MW have been proposed (e.g., Seli et al., 2018). For the purpose of this review, MW will be primarily understood in a broad sense as a drift of attention away from the current situation and task engagement towards unrelated thoughts (Schooler et al., 2011). Specific clinical terms for phenomena related to MW, such as rumination and maladaptive daydreaming, will be used when these terms have been applied in the reviewed articles. These terms will be explained when they are first introduced. For instance, rumination is mainly characterized by its perseverative nature and negative contents, such as negatively biased memories and thoughts about one’s future (Smith and Alloy, 2009). It can be regarded as a subtype of MW (e.g., Van Vugt et al., 2018), but has also been viewed as distinct from MW (e.g., Christoff et al., 2016) or as overlapping with MW (e.g., Linz et al., 2021). While there are arguments for each of these views, within the scope of this review, rumination, daydreaming, as well as repetitive and perserverative thinking will be regarded as subtypes of MW. For further information on clinical terms for phenomena related to MW, readers are referred to more comprehensive reviews on MW in ADHD (Bozhilova et al., 2018), depression (Chaieb et al., 2022), anxiety disorders (Fell et al., 2023), and mental health in general (Kucyi et al., 2023).

The most common approach to measuring MW is through self-report questionnaires (e.g., Mowlem et al., 2019; Mrazek et al., 2013). Experience sampling represents a more elaborate approach, which involves probing the inner state with questions related to the focus of attention and the contents of thoughts (e.g., Hurlburt and Heavey, 2001). Experience sampling is conducted during rest, daily routines or experimental tasks that typically require continuous attention.

Several positive functions of MW have been reported, such as its contributions to creativity, problem solving, autobiographical planning and adaptation to changing situations (e.g., Mooneyham and Schooler, 2013). However, MW has also been linked to lowered mood (e.g., Killingsworth and Gilbert, 2010) and increases in anxiety (e.g., Arch et al., 2021) and stress (e.g., Crosswell et al., 2020). Therefore, it has been suggested that the connection between MW and affect may contribute to the emergence of psychiatric disorders (e.g., Fell, 2012). Another significant area of exploration, gaining increasing attention, centers on the relationship between MW and sleep patterns (for an overview, see, e.g., Cárdenas-Egúsquiza and Berntsen, 2022). This conceptual triangle is completed by the extensive research dedicated to exploring the interrelation between sleep and affect.

The findings concerning the interplay between sleep and affect have been scrutinzed in numerous comprehensive review articles. The consensus among most reviews is that the temporal patterns of changes in sleep and affect point to a bidirectional relationship (Altena et al., 2016; Dinis and Braganca, 2018; Kahn et al., 2013; Vandekerckhove and Cluydts, 2010; Vandekerckhove and Wang, 2017; Watling et al., 2016). On one hand, daytime affect and emotional stress have been observed to diminish subsequent subjective and objective sleep quality (e.g., Altena et al., 2016; Kahn et al., 2013; Krizan et al., 2023; Vandekerckhove and Wang, 2017). Conversely, the quality of sleep appears to impact subsequent daytime affect and the reactivity to emotional events (e.g., Altena et al., 2016; Kahn et al., 2013; Pilcher and Huffcutt, 1996; Vandekerckhove and Wang, 2017).

Assuming a bidirectional relationship between sleep and affect, this brief review aims to focus on the relation between MW and affect, as well as MW and sleep. The review will concentrate on MW propensity rather than other characteristics, such as its content. Should evidence also suggest bidirectional influences, the dynamics between MW, affect, and sleep could be construed as a threefold vicious cycle. However, the bulk of research exploring the connection between MW and affect (for an overview, see, e.g., Fox et al., 2018), as well as MW and sleep (Cárdenas-Egúsquiza and Berntsen, 2022) is primarily correlational, leaving the direction of the influences unclear. Therefore, the limited studies that have directly explored potential directional relationships will be described below (for an overview, see Table 1).

**Table 1:** Summary of the studies discussed in this mini-review.

Authors	Year	Title	Sample size	Age (years)	Intervention	Data: experience sampling (ES), self-report questionnaires (SRQ), etc.	Key finding(s)
Seibert and Ellis	1991	Irrelevant thought, emotional mood states, and cognitive task performance	*N* = 90	Not specified (introductory students)	Mood induction (positive, neutral, negative)	ES of MW during and after a memory task	More MW after positive and negative versus neutral mood induction
Guastella and Moulds	2007	The impact of rumination on sleep quality following a stressful life event	*N* = 114	Not specified (third-year students)	Pre-sleep rumination versus distraction condition	SRQ for rumination, intrusions, sleep quality	More pre-sleep intrusive thoughts and poorer subsequent sleep quality in subjects with high trait rumination
Smallwood et al.	2009	Shifting moods, wandering minds: negative moods lead the mind to wander	*N* = 25	21.7 ± 2.0 (mean ± s.d.)	Mood induction (positive, neutral, negative)	SRQ for MW after sustained attention task	More attentional lapses and MW after negative versus positive mood induction
Zoccola et al.	2009	Rumination predicts longer sleep onset latency after an acute psychosocial stressor	*N* = 70	19.5 ± 1.5 (mean ± s.d.)	Stress induction (delivering a short speech)	SRQ for trait and state rumination, sleep; actigraphy	Both state and trait rumination predicted longer subsequent subjective and objective sleep onset latency
Killingsworth and Gilbert	2010	A wandering mind is an unhappy mind	*N* = 2,250	34.0 (mean)		App-based daily-life ES of MW and mood	Enhanced MW followed by negative mood
Marchetti et al.	2012	Mindwandering heightens the accessibility of negative relative to positive thought	*N* = 79	20.3 ± 2.6 (mean ± s.d.)		ES of MW during a sustained attention task; SRQ for depression, rumination, affect; test for accessibility of negative cognitions	Increased MW predicted higher accessibility of negative thoughts in subjects with elevated depression scores
Ottaviani and Couyoumdjian	2013	Pros and cons of a wandering mind: a prospective study	*N* = 40	24.5 ± 4.9 (mean ± s.d.)		ES of MW during online tracking task; ES of MW across day; SRQ for sleep; ambulatory electrocardiography	Higher frequency of MW associated with enhanced heart rate and increased difficulty falling asleep
Poerio et al.	2013	Mind-wandering and negative mood: does one thing really lead to another?	*N* = 24	24.17 ± 2.9 (mean ± s.d.)		App-based daily-life ES of MW and mood	No association between amount of MW and later sadness and anxiety; increased sadness followed by enhanced MW
Ruby et al.	2013	How self-generated thought shapes mood – the relation between mind-wandering and mood depends on the socio-temporal content of thoughts	*N* = 58	25.5 (mean; range 21–31)		ES of MW and mood during a sustained attention task	Heightened levels of MW led to decreased mood when mood was initially high; lower mood predicted enhanced subsequent MW
Stawarczyk et al.	2013	Concern-induced negative affect is associated with the occurrence and content of mind-wandering	*N* = 32	Subgroup1: 21.75 ± 2.24 Subgroup2: 22.44 ± 3.50 (mean ± s.d.)	Induction of concerns about a negative future event versus neutral event anticipation	ES of MW during a sustained attention task; SRQ for positive and negative affect	Increased negative affect following concern induction predicted greater MW propensity
Pillai et al.	2014	A seven day actigraphy-based study of rumination and sleep disturbance among young adults with depressive symptoms	*N* = 42	19.55 ± 3.20 (mean ± s.d.)		SRQ for sleep, depression, rumination; actigraphy	Increased pre-sleep rumination predictive of longer subjective and objective sleep onset latency, but not of total sleep time and sleep efficiency
Takano et al.	2014	Repetitive thought impairs sleep quality: an experience sampling study	*N* = 43	19.4 ± 1.3 (mean ± s.d.)		App-based daily-life ES of MW; SRQ for positive and negative affect; actigraphy	Repetitive evening thought associated with longer sleep onset latency, reduced sleep efficiency and total sleep time; reduced sleep efficiency and total sleep time predicted reduced positive affect the next day
Poh et al.	2016	Sleepless night, restless mind: effects of sleep deprivation on mind wandering	*N* = 48	Subgroup1: 23.87 Subgroup2: 22.43 (mean)	Total sleep deprivation versus regular night	ES of MW during a visual search task; SRQ for sleep; actigraphy	More MW and less meta-awareness of MW in sleep deprived subjects versus controls
Van Laethem et al.	2016	Day-to-day relations between stess and sleep and the mediating role of perseverative cognition	*N* = 44	35.0 ± 10.1 (mean ± s.d.)		SRQ for sleep, stress, perseverative cognition; actigraphy	Higher daily stress associated with more daily perseverative thoughts, which predicted lower sleep quality and sleep efficiency
Walter and Trick	2018	Mind-wandering while driving: the impact of fatigue, task length, and sustained attention abilities	*N* = 40	18.7 (mean)		ES of MW during a simulator driving task; SRQ for sleep; sustained attention task	Number of hours slept the previous night best advance predictor of MW
Marcusson-Clavertz et al.	2019	A daily diary study on maladaptive daydreaming, mind wandering, and sleep disturbances: examining within-person and between-persons relations	*N* = 126	30.04 ± 10.65 (mean ± s.d.)		SRQ for MW, maladaptive daydreaming, sleep	Subjective sleep disturbances predicted MW, but not maladaptive daydreaming the next day; MW and daydreaming did not predict sleep disturbances the next night
Robison et al.	2020	A multi-faceted approach to understanding individual differences in mind-wandering	*N* = 332	Not specified		ES of MW during six different tasks; SRQ for sleep, affect, daydreaming, MW, mindfulness	MW during tasks did not correlate with subjective sleep duration on the night before
Sladek et al.	2020	Daily rumination about stress, sleep, and diurnal cortisol activity	*N* = 61	20.91 ± 0.36 (mean ± s.d.)		SRQ for sleep, rumination, stress; actigraphy; cortisol measurement	Positive association between daily stress and subsequent objective sleep onset latency on days with elevated rumination
Unsworth et al.	2021	Individual differences in lapses of attention: a latent variable analysis	*N* = 358	Not specified		ES of MW during four different tasks; SRQ for sleep, mindfulness	Prior sleep duration correlated with MW during sustained attention and Stroop, but not during working memory and psychomotor vigilance tasks
Beloborodowa et al.	2023	College students’ daily mind wandering is related to lower social well-being	*N* = 648	Subgroup1: 18.41 ± 0.69 Subgroup2: 18.15 ± 0.39 Subgroup3: 18.62 ± 0.66 (mean ± s.d.)		Online daily-life ES of MW, loneliness, felt connection to others, school belonging	Increased day-to-day MW predicted greater subsequent loneliness and reduced concurrent connection to others and school belonging
Besten et al.	2023	The impact of mood-induction on maladaptive thinking in the vulnerability for depression	*N* = 82	Subgroup1: 19.0 Subgroup2: 20.0 (mean)	Positive fantasizing versus stress induction	ES of MW during a sustained attention task; SRQ for affect, susceptibility to negative affect	Less MW after fantazising versus stress; greater tendency for MW after stress versus fantazising in subjects more susceptible to negative affect
Marcusson-Clavertz et al.	2023	Mind wandering and sleep in daily life: a combined actigraphy and experience sampling study	*N* = 202	30.04 ± 10.65 (mean ± s.d.)		Daily-life ES of MW; SRQ for MW, sleep; actigraphy	Subjective and objective sleep parameters did not predict MW next day; interindividual differences in sleep disturbances and duration associated with amount of unguided thoughts
Simor et al.	2024	Reduced REM and N2 sleep, and lower dream intensity predict increased mind-wandering	*N* = 67	24.7 ± 3.3 (mean ± s.d.)		SRQ for MW, affect, sleep; mobile electroencephalography (EEG) via headband	Objective, but not subjective sleep parameters predictive of MW next day

In these studies, the direction of relationships is inferred from the time points to which the measurements of the properties can be assigned. If these time points are clearly sequential, a direction can be inferred and is often interpreted in terms of causation. For instance, if the measurement of sleep parameters refers to a certain night and MW parameters are measured the following day, a significant correlation between the sleep and MW parameters may be interpreted as sleep influencing MW. However, temporal sequence only provides an indicator of causation, but does not prove it, as famously noted by Hume (1739).

## 2 Interrelation between mind wandering and affect

First, the directional relationship between mind wandering (MW) and affect will be examined. Does intensified MW lead to increases/decreases in negative/positive affect, and vice versa? Concerning the influence of MW on affect, Killingsworth and Gilbert (2010) analyzed data from 2,250 subjects collected via app-based daily-life experience sampling. Based on multi-level regression analyses, they reported that overall MW was associated with diminished mood, and a statistically significant decrease in mood was observed even for neutral MW content. Importantly, time-lag analyses strongly suggested that negative mood followed enhanced MW.

Based on a similar approach, Poerio et al. (2013) collected data from a much smaller group of 24 subjects. They did not find a significant association between the overall amount of MW and later feelings of sadness or anxiety. However, sad and anxious MW content predicted subsequent sadness and anxiety. Furthermore, increased sadness was followed by heightened levels of MW.

Marchetti et al. (2012) assessed MW and its emotional valence in 79 students using experience sampling during a sustained attention task. Moreover, depression symptoms were examined and accessibility of negative cognitions was measured before and after the task via a neuropsychological test (scrambled sentences task, Van der Does, 2005). Based on hierarchical regression analyses, the authors reported that increased MW during the attentional task predicted higher accessibility of negative thoughts after the task in subjects with elevated levels of depression symptoms.

Beloborodova et al. (2023) investigated 648 students through experience sampling using electronic survey software. Social well-being in terms of loneliness, connection to others, and school belonging, as well as MW were evaluated over the span of two academic quarters or an academic semester. Increased day-to-day MW predicted greater loneliness at the next time point and was concurrently associated with reduced feelings of connection to others and lower school belonging. Furthermore, thoughts focused on the past and future were linked to lower social well-being compared to present-oriented thoughts.

With regard to the influence of affect on MW, Seibert and Ellis (1991) explored MW in 90 students who performed a memory task, collecting data through verbal reports during the task and written reports afterward. Before the task, subjects underwent mood induction procedures to induce either positive, neutral, or negative moods. These procedures involved the presentation of 25 self-referent statements with positive, neutral, or negative content. In both types of reports, subjects described experiencing more instances of MW following both positive and negative mood inductions compared to neutral mood induction.

Smallwood et al. (2009) induced positive, neural, and negative moods in 25 subjects by presenting emotionally valenced video clips. Subjects then engaged in a sustained attention task and subsequently completed a MW questionnaire. Compared to a positive mood, the induction of a negative mood was followed by more attentional lapses and by increased MW. Moreover, negative mood led to a greater tendency for past-related MW (Smallwood and O’Connor, 2011).

Besten et al. (2023) investigated MW during a sustained attention task in 82 university students, who were categorized into two subgroups based on their susceptibility to negative affect. Prior to the task, subjects underwent a positive fantasizing or a stress induction session. After engaging in fantasizing, subjects experienced less MW and thoughts were found to be less past-related and negative compared to after experiencing stress. Subjects more susceptible to negative affect exhibited a greater tendency for MW following stress versus fantasizing compared to those less susceptible to negative affect.

Regarding a bidirectional relation between MW and affect, Stawarczyk et al. (2013) examined 32 subjects using experience sampling during a sustained attention task. Prior to the task, they induced either concerns about a negative future event or a neutral event anticipation. The authors found that increased negative affect following concern induction predicted a greater propensity for MW. Moreover, negative affect decreased to a lesser degree over time in subjects who reported more MW related to the induced concern.

Ruby et al. (2013) assessed MW and mood in 58 subjects using experience sampling during a task requiring sustained attention. Through linear mixed models and lag analyses, they discovered that heightened levels of MW led to decreased mood when mood was initially high, but not when it was low. Negative versus positive thought content strongly correlated with elevated versus diminished subsequent mood. Conversely, lower mood generally predicted enhanced subsequent MW, particularly when initial MW was low.

**Figure 1:**
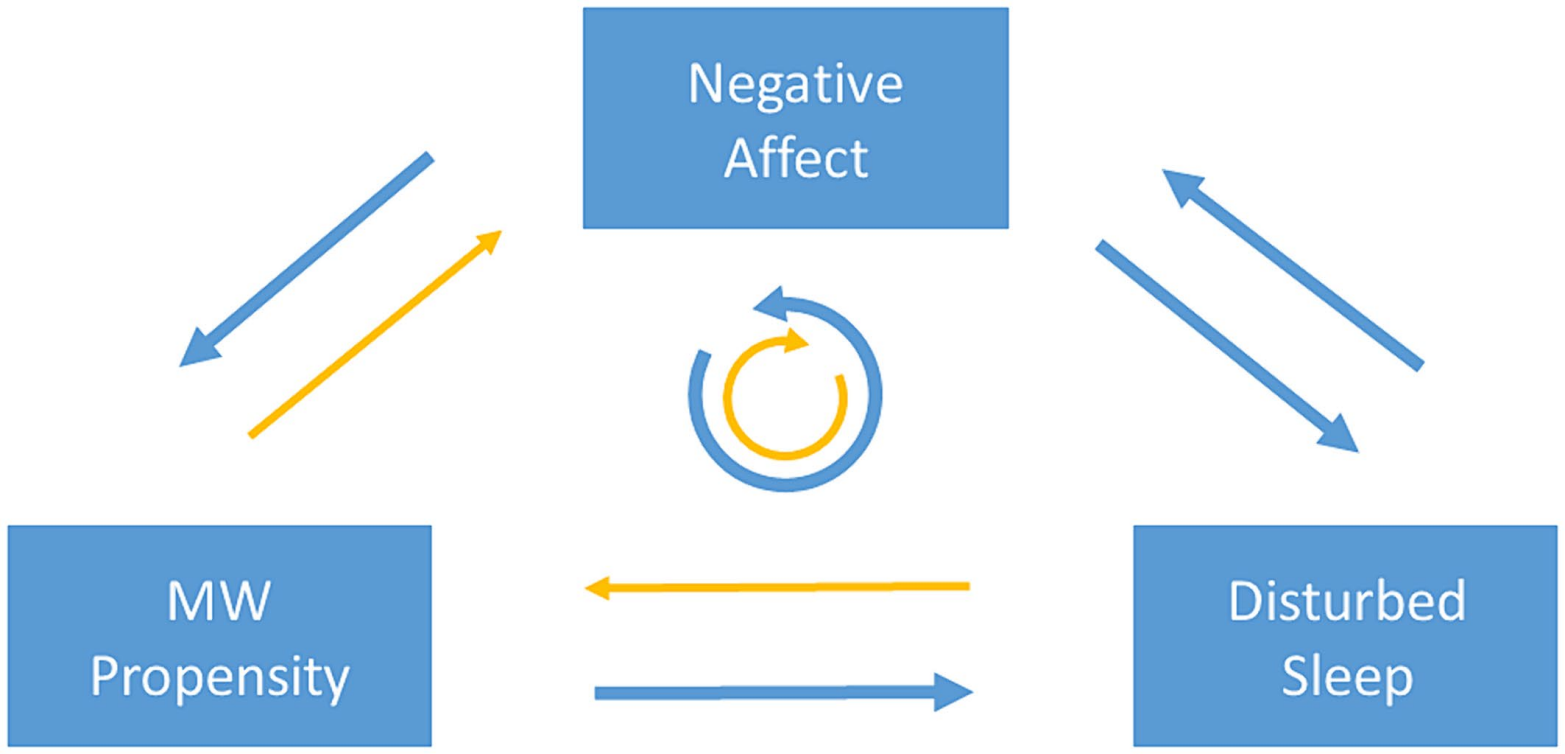
Schematic illustration of the threefold reinforcing cycle between mind wandering, sleep, and affect. Arrows vizualize directional influences: blue and thicker arrows indicate convincing evidence for an influence, while yellow and thinner arrows indicate mixed evidence and a possibly smaller influence. The circles with arrows in the center symbolize possible impact cycles.

Based on these studies, there is unequivocal evidence for an impact of affect on subsequent MW propensity. In particular, increased negative mood appears to be followed by enhanced MW. Results regarding the influence of the amount of MW on subsequent affect are less definitive. It is possible that this effect is less pronounced and only becomes statistically significant when examining large cohorts. As a side note, findings regarding the contents of MW are more conclusive. A distinct relationship seems to exist between negatively versus positively valenced MW and subsequent negative versus positive shifts in affect. Taken together, there is evidence for a reinforcing loop between MW propensity and negative affect, with stronger indications pointing to an influence of affect on MW than vice versa (Figure 1).

## 3 Interrelation between mind wandering and sleep

Secondly, the relationship between MW and sleep will be examined. Cárdenas-Egúsquiza and Berntsen (2022) reviewed evidence for an influence of poor sleep on MW based on examination of 21 studies. They concluded that self-reported disturbed sleep and experimentally induced sleep deprivation are associated with an increase in disruptive MW, i.e., MW that is detrimental for task performance and goal achievement. However, they also pointed out that most of the available data are correlational and that the causal direction of the relationship between sleep and MW is still unclear. Below, studies will be discussed that suggest temporally sequential influences of MW on sleep, or vice versa.

Regarding the influence of MW on sleep, Zoccola et al. (2009) investigated the relationship between rumination, i.e., a pathological type of MW characterized by negative and perseverative thoughts, and subsequent sleep onset latency in a sample of 70 subjects. As a stressor, subjects were required to deliver a short speech in front of a panel, and questionnaires assessed trait rumination before and state rumination after the speech. The authors found that both state and trait rumination predicted longer subsequent subjective and actigraphy-based sleep onset latency. There were no significant effects on sleep duration and wake time after sleep onset.

Ottaviani and Couyoumdjian (2013) explored MW in 40 subjects by employing thought probes during an online tracking task and through ambulatory electrocardiography (ECG) and a 24-h electronic diary. They observed that a higher frequency of MW was associated with an enhanced 24-h heart rate and increased subjective difficulty falling asleep the following night.

Guastella and Moulds (2007) assessed self-reported sleep quality in 114 students following a stressful event (mid-term exam). The students were divided into low and high trait rumination subgroups and were randomly assigned to either a pre-sleep rumination or distraction condition. The study revealed that high-trait rumination individuals reported more pre-sleep intrusive thoughts and poorer subsequent sleep quality.

Pillai et al. (2014) examined self-reported rumination and sleep across 1 week in 42 students with at least moderate levels of depressive symptoms. Increased pre-sleep rumination was predictive of longer self-reported and actigraphy-based sleep onset latency, but not of total sleep time and sleep efficiency (total sleep time divided by time in bed).

Sladek et al. (2020) investigated self-reported rumination, stress, cortisol levels and sleep in 61 subjects across 8 nights. Across subjects, greater daily stress levels were accompanied by higher waking cortisol levels and shorter sleep duration. Within subjects, daily stress did not directly predict longer actigraphy-based sleep onset latency. However, a trend suggested that the interaction between daily stress and daily rumination predicts sleep onset latency. Exploring this trend, a positive association between daily stress and subsequent sleep onset latency was observed on days with elevated rumination levels.

Van Laethem et al. (2016) assessed 44 PhD students over a two-month period, encompassing their thesis defense, utilizing self-reports and actigraphy. They found that MW in the form of perseverative thoughts acted as a day-level mediator between stress and both subjective sleep quality and objective sleep efficiency. In other words, higher daily stress was associated with more daily perseverative thoughts, which, in turn, predicted lower sleep quality and reduced sleep efficiency.

Concerning the impact of sleep on MW, Marcusson-Clavertz et al. (2023) collected actigraphy measurements, answers to thought probes and questionnaire data in 202 subjects across 7 days and 8 nights. MW was differentiated into task-unrelated, stimulus-independent, and unguided thoughts. Subjective and objective sleep parameters did not predict MW the following day, either within or between subjects. However, between-subject differences in sleep disturbances and duration were associated with the amount of unguided thoughts.

Robison et al. (2020) explored MW in 332 subjects by employing thought probes during a battery of tasks and through various questionnaires. They found that MW propensity during the tasks did not significantly correlate with questionnaire-based sleep duration on the night before. As a side note, the implemented approach of inquiring about sleep the previous night at the time of task performance may be considered suboptimal for analyzing the temporal relationship between sleep and MW.

Using a similar approach involving thought probes and questionnaires, Unsworth et al. (2021) investigated lapses of attention and MW in 358 subjects. They observed that MW significantly correlated with sleep duration on the prior night for a sustained attention to reponse (SART) and Stroop task, but not for a working memory and psychomotor vigilance task.

Simor et al. (2024) examined 67 subjects in their home environment using mobile sleep EEG headbands and self-report scales over a week. Their findings indicated that objective, but not subjective sleep parameters, were predictive of MW the following day. Specifically, reductions in N2 and REM sleep duration predicted increased subsequent MW.

Walker and Trick (2018) explored MW in 40 subjects using thought probing during a driving task in a simulator. They compared several self-report and SART performance variables and concluded that the number of hours slept the previous night was the best advance predictor of MW.

Poh et al. (2016) investigated 48 subjects, who were randomly assigned to either a subgroup with a regular night in the sleep lab or a subgroup experiencing total sleep deprivation. Based on thought probes administered during a visual search task conducted the following day, sleep deprived subjects reported more MW and less meta-awareness of MW (i.e., awareness of the drift of attention away from the current task and situation) compared to control subjects.

With regard to a bidirectional relationship between MW and sleep, Marcusson-Clavertz et al. (2019) examined 126 subjects experiencing self-identified maladaptive daydreaming (i.e., a very immersive and intense form of MW, often involving vivid, elaborate and emotionally engaging scenarios). Through questionnaires completed on up to 8 consecutive days, they discovered that sleep disturbances predicted MW the following day, but not maladaptive daydreaming. Maladaptive daydreaming and MW did not significantly predict subjective sleep disturbances the subsequent night. However, the validity of these findings is limited by the fact that questionnaires were completed only once per day in the evening (after 6 pm).

Takano et al. (2014) assessed self-reported thought content, mood and actigraphy-based sleep parameters in 43 students during a one-week period. They found that repetitive thought in the evening was associated with increased sleep onset latency, as well as reduced sleep efficiency and total sleep time in the following night. In turn, reduced sleep efficiency and total sleep time predicted reduced positive affect on the next day. Moreover, reduced positive affect and increased repetitive thoughts on the next morning were correlated and repetitive thought was autocorrelated across the day.

To summarize, the majority of studies suggest that increased MW, in particular in the form of repetitive thoughts resembling rumination, has an influence on subsequent sleep. While some studies did find an effect on sleep duration and efficiency and others did not, most studies report an influence on sleep onset latency or difficulty falling asleep. Evidence for an impact of sleep on subsequent MW is less clear. Available studies suggest that this impact may depend on the situation in which MW is assessed and whether subjective or objective sleep measures are considered. Taken together, there is also evidence for a reinforcing loop between MW propensity and disturbed sleep, with stronger indications pointing to an influence of MW on sleep than vice versa (Figure 1).

## 4 Discussion

To conclude, available data support the idea of feedback cycles between MW and sleep, sleep and affect, as well as affect and MW. Therefore, one may assert that these individual loops constitute a threefold vicious cycle, which may contribute to the development and perpetuation of psychiatric disorders, such as depression (e.g., Chaieb et al., 2022), anxiety disorders (e.g., Fell et al., 2023), and ADHD (Bozhilova et al., 2018). In terms of equivocal findings across studies, available data provide more convincing evidence for an impact cycle in the direction “MW propensity → disturbed sleep → negative affect → MW propensity” than in the inverse direction (Figure 1). Based on this notion and speculatively speaking, interventions targeting disturbed sleep may be more effective in improving affect than those focusing solely on excessive MW. Ideally, however, both excessive MW and disturbed sleep should be addressed clinically, and both aspects should be monitored.

Different methods were applied to measure MW, affect, and sleep. Experience sampling-based measures of MW and affect may be regarded as more reliable than self-report questionnaires because the latter depend more on self-image and the ability to self-reflect (e.g., Belardi et al., 2022). Objective measures of sleep, such as polysomnography and actigraphy, are generally considered more reliable than subjective measures based on self-report. However, subjective measures are more widely accessible and also relevant, for instance, to quality of life and daily functioning (e.g., Fabbri et al., 2021). As shown in the study by Simor et al. (2024), where objective but not subjective sleep parameters were predictive of subsequent MW, findings based on objective versus subjective sleep measures may diverge.

Finally, it should be noted that this brief review primarily focused on findings related to the amount of MW and healthy subjects. Certainly, the described dynamics may considerably depend on the studied sample and on predominant characteristics of MW, including its emotional valence, temporal orientation, self-relatedness, deliberateness, disruptiveness, perseveration, and whether it is accompanied by meta-awareness. For instance, MW in depression and anxiety disorders is more negative and perseverative than in controls and tends to be more past-related in depression and more future-related in anxiety disorders (Chaieb et al., 2022; Fell et al., 2023). Moreover, it has been suggested that the impact of MW on affect depends on the presence of meta-awareness (Konjedi and Maleeh, 2017). Further research is necessary to unravel the consequences of a modulation of the propensity and characteristics of MW on sleep and affect across different diagnostic groups and individuals. Interventions aimed at both reducing the frequency of MW and modifying its characteristics may be necessary to interrupt the vicious cycle between MW, poor sleep and negative affect.
